# ULSOSEAL Technique: A Unique Technique to Achieve Hemostasis Using ExoSeal in High-Risk Patients after Common Femoral Artery Puncture

**DOI:** 10.1155/2021/2470333

**Published:** 2021-08-28

**Authors:** Yohsuke Honda, Shinsuke Mori, Tomoya Fukagawa, Toshihiko Kishida, Takahide Nakano, Shigemitsu Shirai, Masafumi Mizusawa, Kenji Makino, Masakazu Tsutsumi, Norihiro Kobayashi, Masahiro Yamawaki, Yoshiaki Ito

**Affiliations:** Department of Cardiology, Saiseikai Yokohama City Eastern Hospital, Yokohama-Shi, Japan

## Abstract

This study aimed to assess the safety and feasibility of the ULtrasound-guided uSe Of exoSEAL technique (ULSOSEAL technique) in patients at a high risk of complications following the use of ExoSeal. ExoSeal is a novel, completely extravascular hemostatic device that can treat punctures of the common femoral artery; however, it is not preferable for use in cases that require hemostasis of complex puncture sites. From November 2019 to August 2020, the ULSOSEAL technique was performed in 35 patients with implanted stents (6 patients, 17%), severe calcification (32 patients, 91%), and plaque (7 patients, 20%) around the puncture site; the presence of these conditions is usually undesirable when using ExoSeal. The antegrade approach was used in 22 patients (71%). The size of the ExoSeal used was 5 Fr (13 patients, 37%), 6 Fr (21 patients, 60%), and 7 Fr (1 patient, 2%). Technical success was achieved in 34 patients (97%), while ExoSeal malfunction occurred in 1 patient. There was no incidence of vessel occlusion, pseudoaneurysm, arteriovenous fistula, infection, and secondary bleeding. One patient developed a hematoma (>5 cm in size); however, it occurred before the use of ExoSeal due to side leakage from the inserted sheath. The ULSOSEAL technique was safe and feasible for hemostasis in patients who were considered unsuitable for the ExoSeal device.

## 1. Introduction

It is widely known that vascular closure devices (VCDs) are noninferior to manual compression for managing vascular access site complications and reducing the time to hemostasis in patients undergoing transfemoral catheter intervention [1, 2]. In the past decade, various VCDs have become available for the management of access site complications following percutaneous vascular interventions [3]. The ExoSeal VCD (Cordis Corporation, Miami Lakes, Florida) is a novel bioabsorbable device that can seal the sites of femoral artery puncture in patients undergoing transfemoral interventional procedures. ExoSeal is a completely extravascular device that reduces the risk of anchor-related luminal narrowing or occlusion, adverse effects on the deep femoral artery, and infection. ExoSeal use is safe and painless, with an excellent rate of technical success during the antegrade approach, even in cases with severe calcification of the access vessel [4]. A 7-Fr ExoSeal vascular closure device was reported to be safe and effective in closing femoral puncture sites created by size 8- or 9-Fr introducer sheaths [5]. Thus, ExoSeal is a VCD with potential for wider use.

However, there are peculiar complications associated with ExoSeal use, such as distal embolization of the plug material due to the procedure while fixing the device [6]. Moreover, the use of ExoSeal is associated with a higher complication rate than with the use of collagen-based sutures or medicated devices [7]. We conjectured that the presence of structures projecting into the luminal cavity, such as implanted stents, severe calcification, and plaques around the puncture site, could interfere with the optimal maneuverability of ExoSeal and increase the risk of complications. Patients with such conditions, who are considered to have a high risk of complications following the use of ExoSeal, were excluded from a previous study [1]. Our clinical experience indicates that the use of real-time ultrasound could clearly guide ExoSeal insertion. In this study, we evaluated the safety and feasibility of the real-time ULtrasound-guided uSe Of exoSEAL technique (ULSOSEAL technique) in patients contraindicated for the deployment of ExoSeal.

## 2. Materials and Methods

### 2.1. Study Population

**Figure 1:**
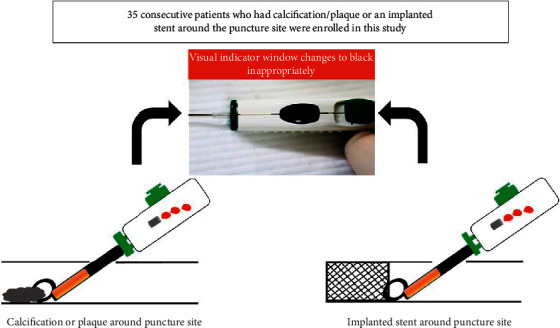
Cases conventionally contraindicated for ExoSeal use.

In this case series report, we investigated the clinical outcomes following the use of the ULSOSEAL technique. From November 2019 to August 2020, the ULSOSEAL technique was performed in 35 patients undergoing endovascular therapy who were considered to have a high risk of complications following the deployment of ExoSeal, in whom the process of inserting ExoSeal was anticipated to be difficult (Figure 1).

Patients at a high risk of complications due to ExoSeal use were defined as those who had at least one of the following: implanted stents, severe calcification, or plaque around the puncture site, which could interfere with ExoSeal deployment.

### 2.2. The ULSOSEAL Technique

**Figure 2:**
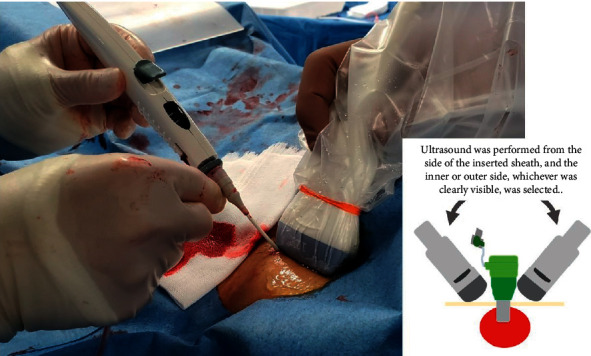
The ULSOSEAL technique.

This technique was performed by two physicians: one physician maneuvered the ExoSeal, and the other performed the sonography (Figure 2).

**Figure 3:**
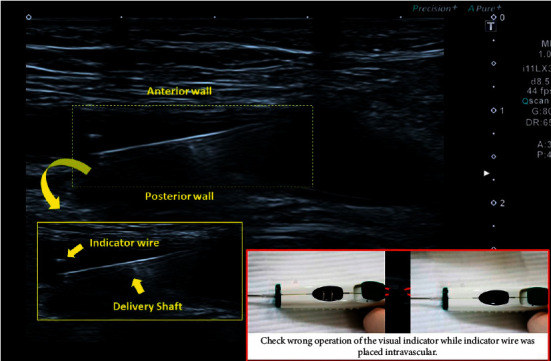
Confirmation of correct placement of the indicator wire.

Ultrasound was performed from the side of the inserted sheath; the inner or outer side, whichever was clearly visible, was selected for imaging. We inserted the ExoSeal into the sheath and retracted the sheath until it was secured to the ExoSeal; thereafter, the indicator wire was deployed. The sheath and ExoSeal were pulled back until the bleed-back indicator showed pulsatile flow. We monitored this process on ultrasound and found that the visual indicator provided correct feedback when the indicator wire was placed intravascularly (Figure 3).

**Figure 4:**
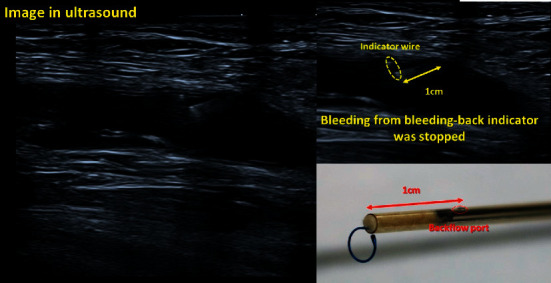
Ultrasound finding observed upon cessation of bleeding in the bleeding-back indicator.

We continued to retract the sheath and ExoSeal, and a significant reduction in pulsatile flow was observed in the bleed-back indicator. The flow from the bleed-back indicator stopped when the backflow port, placed 1 cm from the tip, moved outside the vessel (Figure 4).

**Figure 5:**
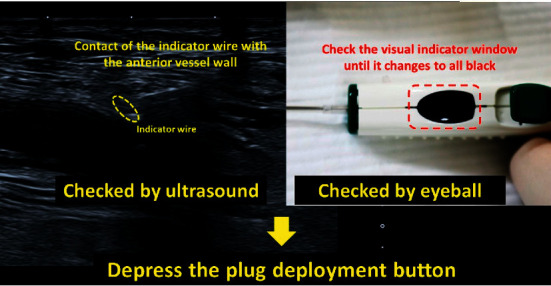
Ultrasound finding when we should depress the plug deployment button.

Using ultrasound, we confirmed that the looped indicator wire was in contact with the anterior wall; we visually monitored the indicator window until it changed to all black, following which, we pressed the plug deployment button (Figure 5).

**Figure 6:**
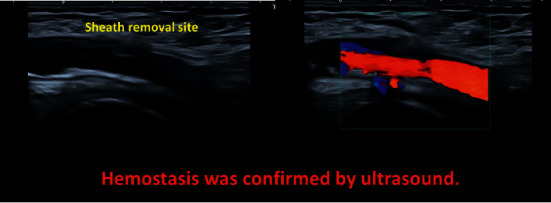
Confirmation of hemostasis by ultrasound.

After the sheath was removed using the ExoSeal, manual compression was performed with an ultrasonic probe for approximately 5 min; subsequently, using ultrasound, we confirmed that complete hemostasis was achieved (Figure 6).

This novel technique required 1-2 minutes, and the duration did not differ from that required for non-ultrasound-guided ExoSeal use.

### 2.3. Interventional Procedure

The selection of the puncture site and the type of endovascular strategy were determined by the operators based on the clinical condition of the patients. In all cases, the duplex ultrasound-guided puncture was performed [8]. When an ipsilateral antegrade femoral approach was employed, a 4.5-Fr parent sheath, 6-Fr sheath, 6-Fr sheathless PV (Asahi Intec, Aichi, Japan), or 6-Fr destination (Terumo, Tokyo, Japan) guide catheter was placed. When the contralateral femoral approach was employed, a 6-Fr sheathless PV, 6-Fr destination guide catheter, or Flexor Ansel (Cook Medical, Bloomington, IN, USA) was used. After inserting the sheath, unfractionated heparin (5,000 units) was routinely administered through the artery, with additional heparin administered intravenously during the procedure to maintain an activated clotting time of >200 s. Antiplatelet therapy included aspirin (100 mg daily) and clopidogrel (75 mg daily) or cilostazol (100 mg twice daily), which was initiated at least 1 week before the endovascular therapy. A 5-Fr ExoSeal was used after removing the 4.5-Fr parent sheath. A 6-Fr ExoSeal was used after removing the 6-Fr sheath, 6-Fr sheathless PV, or 6-Fr destination guide catheter. A 7-Fr ExoSeal was used after Flexor Ansel removal.

### 2.4. Study Endpoint

The primary outcome measure was the technical success. Secondary outcomes were the incidence of adverse events during the periprocedural period and within 30 days after the procedure. Technical success was considered as the deployment of the plug at the puncture site and achievement of hemostasis without complications. Major adverse events were defined as vessel occlusion due to endoluminal deposition of the plug, occurrence of pseudoaneurysms or arteriovenous fistulas, secondary bleeding that required further treatment (i.e., blood transfusion or surgical treatment), and puncture site infection. Minor adverse events were defined as secondary bleeding or hematomas >5 cm in size that did not require further treatment.

## 3. Results

### 3.1. Patient Characteristics

The patient characteristics are shown in Table 1.

The number of patients who underwent hemodialysis was 12 (35%), and 29 patients (83%) received dual antiplatelet therapy. Three patients (9%) received anticoagulant therapy, and the most common drugs used for dual antiplatelet therapy were aspirin and clopidogrel. Overall, 16 patients (48%) developed critical limb ischemia.

### 3.2. Lesion and Procedural Characteristics

Lesion characteristics are shown in Table 2.

The conditions considered as high risk for the occurrence of complications following the use of ExoSeal were as follows: implanted stents (6 patients, 17%), severe calcification (32 patients, 91%), and plaque (7 patients, 20%). Nine patients (26%) had multifactorial disease. The antegrade approach was used in 22 patients (71%). The sizes of the ExoSeal used were 5 Fr in 13 patients (37%), 6 Fr in 21 patients (60%), and 7 Fr in 1 patient (3%).

### 3.3. Periprocedural and 30-Day Incidence of Arterial Access-Related Complications

Perioperative results are presented in Table 3.

Technical success was achieved in 34 patients (97%). Only one case of hematoma of >5 cm in size was observed. One case of failure was considered as malfunction of the ExoSeal because the indicator wire was placed at the anterior wall and the indicator window changed to black; however, we could not press the plug deployment button. In this case, we removed the ExoSeal and quickly switched to manual compression using an ultrasonic probe, and hemostasis was confirmed on ultrasound without any other complications.

One patient developed a hematoma of >5 cm in size; however, it was observed before the deployment of the ExoSeal. We believe that this hematoma was formed by a side leakage around the inserted sheath during the interventional procedure. Failure of the ULSOSEAL technique was not observed in this study cohort.

## 4. Discussion

The main finding of this study is that the ULSOSEAL technique is safe and effective in patients with complications such as implanted stents, severe calcification, or plaque around arterial puncture sites; these are the cases in which the use of ExoSeal is not preferable.

A unique characteristic of the ExoSeal, which is different from other VCDs, is its completely extravascular design and the polyglycolic acid (PGA) plug, which is completely absorbable within 60–90 days. These unique features of ExoSeal could reduce the risk of luminal narrowing or occlusion, harmful effects on the deep femoral artery, and infection, compared with intravascular VCDs. ExoSeal is a VCD that aids manual compression due to its extravascular design, and prolonged manual compression of 2–5 minutes is recommended for hemostasis [9]. In select patients, the use of ExoSeal has been reported to reduce the time to hemostasis and time to ambulation compared to manual compression [1]. However, ExoSeal use was reported to be associated with a higher rate of complications than collagen-based and suture-mediated devices [7].

The complications related to ExoSeal occur because of the deployment of the PGA plug in an inappropriate position: (1) deployment of the PGA plug, not just the extravascular surface of the arterial wall, and (2) deployment of the PGA plug inside the vascular lumen. Manual compression should be initiated in the former case. In case this complication occurs during the ULSOSEAL technique, incomplete hemostasis can be confirmed on ultrasound, and manual compression with an ultrasonic probe can be initiated immediately. In the latter case, manual compression is required, and additional catheter intervention or surgical treatment might be necessary in case of arterial occlusion or acute limb ischemia [6]. The latter is the most critical complication that needs to be avoided during ExoSeal use.

One of the advantages of ExoSeal is the feasibility of removing the sheath quickly, without leaving anything inside the vessel. Unlike extravascular VCDs, hemostasis using ExoSeal was not inferior to manual compression if we could avoid the deployment of the PGA plug inside the vascular lumen.

The major advantage of the ULSOSEAL technique is that we can confirm the position of the indicator wire in real time. We often encounter unforeseen discordance between the position of the indicator wire or the backflow port and visible bleeding through the bleed-back indicator. For example, when both the indicator wire and backflow port were placed inside the vessel, the bleed-back indicator window changed to black. We believe that this occurred due to the structures projecting into the lumen, such as implanted stents, severe calcification, or plaque around the puncture site, which interfere with the appropriate maneuverability of the ExoSeal. Real-time confirmation of the indicator wire using the ULSOSEAL technique was helpful for the use of ExoSeal in such cases with a high risk of complications, and the ULSOSEAL technique could reduce the complications associated with ExoSeal.

Although the ULSOSEAL technique is a noninvasive and cost-free method, a disadvantage is that it requires two physicians for application. However, this technique is not required for all patients and is to be performed only in those at high risk of complications. Another disadvantage of this technique is that severe arterial calcification can interfere with the visibility on ultrasound. The key advantage of this technique is the real-time confirmation of the position of the indicator wire and arterial wall. The ULSOSEAL technique only requires echographical visibility of the puncture site rather than of the entire arterial wall. We performed echo-guided puncture at sites with less calcification, and ultrasound could be performed from the side of the vessel to avoid imaging the calcification sites; hence, we could perform this technique in all patients enrolled in this study. A major limitation of the ULSOSEAL technique is that it cannot be performed in patients whose common femoral artery is not visible on ultrasound at all due to any reasons.

This study has several limitations. First, the sample size was extremely small to evaluate the safety and efficacy of this technique. Second, this was a single-arm study, and a control arm is necessary to confirm the effectiveness of this technique.

## 5. Conclusions

The ULSOSEAL technique was found to be safe and feasible for the use of ExoSeal under ultrasound guidance for hemostasis in patients with implanted stents, severe calcification, or plaque around the puncture site, who were considered to have a high risk of complications following ExoSeal use. Visual confirmation of the intravascular position of ExoSeal and complete hemostasis confirmed using ultrasound are the key highlights of this technique.

## Figures and Tables

**Table 1:** Patient characteristics.

	*n* = 35
Age	75 ± 10
Male, *n* (%)	31 (89)
BMI (kg/m^2^)	23 ± 4
Hypertension, *n* (%)	24 (71)
Diabetes mellitus, *n* (%)	21 (62)
Dyslipidemia, *n* (%)	14 (41)
Smoking, *n* (%)	8 (24)
Hemodialysis, *n* (%)	12 (35)
Cerebrovascular disease, *n* (%)	3 (9)
Coronary artery disease, *n* (%)	15 (45)
Dual antiplatelet therapy, *n* (%)	29 (83)
Anticoagulation, *n* (%)	3 (9)
Critical limb ischemia, *n* (%)	16 (48)

BMI; body mass index.

**Table 2:** Lesion and procedural characteristics.

	*n* = 35
High-risk contraindications for ExoSeal use	
Implanted stent, *n* (%)	6 (17)
Severe calcification, *n* (%)	32 (91)
Plaque, *n* (%)	7 (20)
Target lesion	
Iliac artery, *n* (%)	4 (11)
Common femoral artery, *n* (%)	1 (3)
Superficial femoral artery, *n* (%)	13 (37)
Popliteal artery, *n* (%)	2 (6)
Artery below the knee, *n* (%)	15 (43)
Antegrade puncture, *n* (%)	22 (71)
Size of the used ExoSeal	
5 Fr, *n* (%)	13 (37)
6 Fr, *n* (%)	21 (60)
7 Fr, *n* (%)	1 (3)

**Table 3:** Periprocedural and 30-day incidence of arterial access-related complications.

	*n* = 35
Primary endpoint	
Technical success, *n* (%)	34 (97)
Secondary endpoint to 30 days	
Major adverse events	0 (0)
Vessel occlusion, *n* (%)	0 (0)
Pseudoaneurysm, *n* (%)	0 (0)
Arteriovenous fistula, *n* (%)	0 (0)
Infection, *n* (%)	0 (0)
Minor adverse events	0 (0)
Secondary bleeding, *n* (%)	0 (0)
Hematoma >5 cm, *n* (%)	1 (3)

## Data Availability

Due to the nature of this research, participants of this study did not agree for their data to be shared publicly, so supporting data are not available.
